# RETRACTION: KCTD12 Promotes G1/S Transition of Breast Cancer Cell through Activating the AKT/FOXO1 Signaling

**DOI:** 10.1002/jcla.70219

**Published:** 2026-04-07

## Abstract

**RETRACTION**: . YeR.‐y , . KuangX.‐y , . ZengH.‐j , . ShaoN , . LinY , and . WangS.‐m , “KCTD12 Promotes G1/S Transition of Breast Cancer Cell through Activating the AKT/FOXO1 Signaling,” Journal of Clinical Laboratory Analysis 34, no. 8 (2020): e23315, 10.1002/jcla.23315.32207860 PMC7439418

The above article, published online on 24 March 2020 in Wiley Online Library (wileyonlinelibrary.com), has been retracted by agreement between the journal Editor‐in‐Chief, Rong Fu; and Wiley Periodicals LLC. A third party raised concerns on PubPeer [1] that the sh#1 image in Figure 4E had later been duplicated in another article by different authors as well as its erratum [Sheng et al. 2021 (https://doi.org/10.21037/atm‐21‐1069) and (https://doi.org/10.21037/atm‐2022‐43)]. The third party also reported that the sh#2 image in Figure 4E had been duplicated in another article byanother author group [Peng et al. 2022 (https://doi.org/10.1155/2022/9631036)]. While both of these articles were published subsequently, the images include rotations and additional field of view which indicate the data were derived from the same images, though each article describes different experimental conditions.

The authors responded to an inquiry by the publisher, but the explanation and original data provided did not adequately explain how data had been inadvertently shared between all three author groups. The retraction has been agreed to because the evidence of data shared between three articles and the lack of a sufficient explanation has compromised the editors’ confidence that the results presented in this article are accurate. The authors disagree with the retraction.

References:

[1] AquariusRené , Comments on “KCTD12 promotes G1/S transition of breast cancer cell through activating the AKT/FOXO1 signaling,” PubPeer, August 2024. https://pubpeer.com/publications/B624C6A5FC4650FE0F16C93DDBF4D9.

**RETRACTION**: R.‐y. Ye , X.‐y. Kuang , H.‐j. Zeng , N. Shao , Y. Lin , and S.‐m. Wang , “KCTD12 Promotes G1/S Transition of Breast Cancer Cell through Activating the AKT/FOXO1 Signaling,” Journal of Clinical Laboratory Analysis 34, no. 8 (2020): e23315, 10.1002/jcla.23315.32207860 PMC7439418

The above article, published online on 24 March 2020 in Wiley Online Library (wileyonlinelibrary.com), has been retracted by agreement between the journal Editor‐in‐Chief, Rong Fu; and Wiley Periodicals LLC. A third party raised concerns on PubPeer [1] that the sh#1 image in Figure 4E had later been duplicated in another article by different authors as well as its erratum [Sheng et al. 2021 (https://doi.org/10.21037/atm‐21‐1069) and (https://doi.org/10.21037/atm‐2022‐43)]. The third party also reported that the sh#2 image in Figure 4E had been duplicated in another article byanother author group [Peng et al. 2022 (https://doi.org/10.1155/2022/9631036)]. While both of these articles were published subsequently, the images include rotations and additional field of view which indicate the data were derived from the same images, though each article describes different experimental conditions.

The authors responded to an inquiry by the publisher, but the explanation and original data provided did not adequately explain how data had been inadvertently shared between all three author groups. The retraction has been agreed to because the evidence of data shared between three articles and the lack of a sufficient explanation has compromised the editors’ confidence that the results presented in this article are accurate. The authors disagree with the retraction.

References:

[1] René Aquarius , Comments on “KCTD12 promotes G1/S transition of breast cancer cell through activating the AKT/FOXO1 signaling,” PubPeer, August 2024. https://pubpeer.com/publications/B624C6A5FC4650FE0F16C93DDBF4D9.

